# Identifying associations between diabetes and acute respiratory distress syndrome in patients with acute hypoxemic respiratory failure: an analysis of the LUNG SAFE database

**DOI:** 10.1186/s13054-018-2158-y

**Published:** 2018-10-27

**Authors:** Andrew J. Boyle, Fabiana Madotto, John G. Laffey, Giacomo Bellani, Tài Pham, Antonio Pesenti, B. Taylor Thompson, Cecilia M. O’Kane, Adam M. Deane, Daniel F. McAuley, on behalf of the LUNG SAFE Investigators, Fernando Rios, Frank Van Haren, Mohammad Omar Faruq, T. Sottiaux, P. Depuydt, Fredy S. Lora, Luciano Cesar Azevedo, Eddy Fan, Guillermo Bugedo, Haibo Qiu, Marcos Gonzalez, Juan Silesky, Vladimir Cerny, Jonas Nielsen, Manuel Jibaja, Tài Pham, Hermann Wrigge, Dimitrios Matamis, Jorge Luis Ranero, Charles Gomersall, Pravin Amin, S. M. Hashemian, Kevin Clarkson, Giacomo Bellani, Kiyoyasu Kurahashi, Younsuck Koh, Asisclo Villagomez, Amine Ali Zeggwagh, Leo M. Heunks, Jon Henrik Laake, Waqar Kashif, Jorge Synclair, Jose Emmanuel Palo, Antero do Vale Fernandes, Dorel Sandesc, Yaasen Arabi, Vesna Bumbasierevic, Nicolas Nin, Jose A. Lorente, Anders Larsson, Lise Piquilloud, Boonsong Patjanasoontorn, Fekri Abroug, Daniel F. McAuley, Lia McNamee, Javier Hurtado, Ed Bajwa, Gabriel Démpaire, Guy M Francois, Francesca Rabboni, Sara Conti, Hektor Sula, Alma Cani, Alan Zazu, Christian Dellera, Risso V Alejandro, Julio Daldin, Ruben O Fernandez, Luis P Cardonnet, Lisandro R Bettini, Mariano Carboni Bisso, Emilio M Osman, Mariano G Setten, Pablo Lovazzano, Javier Alvarez, Veronica Villar, Norberto C Pozo, Nicolas Grubissich, Gustavo A Plotnikow, Daniela N Vasquez, Santiago Ilutovich, Norberto Tiribelli, Ariel Chena, Carlos A Pellegrini, María G Saenz, Elisa Estenssoro, Matias Brizuela, Hernan Gianinetto, Pablo E Gomez, Valeria I Cerrato, Marco G Bezzi, Silvina A Borello, Flavia A Loiacono, Adriana M Fernandez, Serena Knowles, Claire Reynolds, Deborah M Inskip, Jennene J Miller, Jing Kong, Christina Whitehead, Shailesh Bihari, Aylin Seven, Amanda Krstevski, Helen J Rodgers, Rebecca T Millar, Toni E Mckenna, Irene M Bailey, Gabrielle C Hanlon, Anders Aneman, Joan M Lynch, Raman Azad, John Neal, Paul W Woods, Brigit L Roberts, Mark R Kol, Helen S Wong, Katharina C Riss, Thomas Staudinger, Xavier Wittebole, Pierre A Bulpa, Alain M Dive, Rik Verstraete, Herve Lebbinck, Pieter Depuydt, Joris Vermassen, Meersseman Philippe, Helga Ceunen, Jonas I Rosa, Daniel O Beraldo, Claudio Piras, Adenilton M Rampinelli, Antonio P Nassar, Sergio Mataloun, Marcelo Moock, Marlus M Thompson, Claudio H Gonçalves, Ana Carolina P Antônio, Aline Ascoli, Rodrigo S Biondi, Danielle C Fontenele, Danielle Nobrega, Vanessa M Sales, Suresh Shindhe, Dk Maizatul Aiman B Pg Hj Ismail, John Laffey, Francois Beloncle, Kyle G Davies, Rob Cirone, Venika Manoharan, Mehvish Ismail, Ewan C Goligher, Mandeep Jassal, Niall D Ferguson, Erin Nishikawa, Areej Javeed, Gerard Curley, Nuttapol Rittayamai, Matteo Parotto, Sangeeta Mehta, Jenny Knoll, Antoine Pronovost, Sergio Canestrini Alejandro R Bruhn, Patricio H Garcia, Felipe A Aliaga, Pamela A Farías, Jacob S Yumha, Claudia A Ortiz, Javier E Salas, Alejandro A Saez, Luis D Vega, Eduardo F Labarca, Felipe T Martinez, Nicolás G Carreño, Pilar Lora, Haitao Liu, Haibo Qiu, Ling Liu, Rui Tang, Xiaoming Luo, Youzhong An, Huiying Zhao, Yan Gao, Zhe Zhai, Zheng L Ye, Wei Wang, Wenwen Li, Qingdong Li, Ruiqiang Zheng, Wenkui Yu, Juanhong Shen, Xinyu Li, Tao Yu, Weihua Lu, Ya Q Wu, Xiao B Huang, Zhenyang He, Yuanhua Lu, Hui Han, Fan Zhang, Renhua Sun, Hua X Wang, Shu H Qin, Bao H Zhu, Jun Zhao, Jian Liu, Bin Li, Jing L Liu, Fa C Zhou, Qiong J Li, Xing Y Zhang, Zhou Li-Xin, Qiang Xin-Hua, Liangyan Jiang, Yuan N Gao, Xian Y Zhao, Yuan Y Li, Xiao L Li, Chunting Wang, Qingchun Yao, Rongguo Yu, Kai Chen, Huanzhang Shao, Bingyu Qin, Qing Q Huang, Wei H Zhu, Ai Y Hang, Ma X Hua, Yimin Li, Yonghao Xu, Yu D Di, Long L Ling, Tie H Qin, Shou H Wang, Junping Qin, Yi Han, Suming Zhou, Monica P Vargas, Juan I Silesky Jimenez, Manuel A González Rojas, Jaime E Solis-Quesada, Christian M Ramirez-Alfaro, Jan Máca, Peter Sklienka, Jakob Gjedsted, Aage Christiansen, Rigshopitalet, Jonas Nielsen, Boris G Villamagua, Miguel Llano, Philippe Burtin, Gautier Buzancais, Pascal Beuret, Nicolas Pelletier, Satar Mortaza, Alain Mercat, Jonathan Chelly, Sébastien Jochmans, Nicolas Terzi, Cédric Daubin, Guillaume Carteaux, Nicolas de Prost, Jean-Daniel Chiche, Fabrice Daviaud, Tài Pham, Muriel Fartoukh, Guillaume Barberet, Jerome Biehler, Jean Dellamonica, Denis Doyen, Jean-Michel Arnal, Anais Briquet, Fanny Klasen, Laurent Papazian, Arnaud Follin, Damien Roux, Jonathan Messika, Evangelos Kalaitzis, Laurence Dangers, Alain Combes, Siu-Ming Au, Gaetan Béduneau, Dorothée Carpentier, Elie H Zogheib, Herve Dupont, Sylvie Ricome, Francesco L Santoli, Sebastien L Besset, Philippe Michel, Bruno Gelée, Pierre-Eric Danin, Bernard Goubaux, Philippe J Crova, Nga T Phan, Frantz Berkelmans, Julio C Badie, Romain Tapponnier, Josette Gally, Samy Khebbeb, Jean-Etienne Herbrecht, Francis Schneider, Pierre-Louis M Declercq, Jean-Philippe Rigaud, Jacques Duranteau, Anatole Harrois, Russell Chabanne, Julien Marin, Jean-Michel Constantin, Sandrine Thibault, Mohammed Ghazi, Messabi Boukhazna, Salem Ould Zein, Jack R Richecoeur, Daniele M Combaux, Fabien Grelon, Charlene Le Moal, Elise P Sauvadet, Adrien Robine, Virginie Lemiale, Danielle Reuter, Martin Dres, Alexandre Demoule, Dany Goldgran-Toledano, Loredana Baboi, Claude Guérin, Ralph Lohner, Jens Kraßler, Susanne Schäfer, Kai D Zacharowski, Patrick Meybohm, Andreas W Reske, Philipp Simon, Hans-Bernd F Hopf, Michael Schuetz, Thomas Baltus, Metaxia N Papanikolaou, Theonymfi G Papavasilopoulou, Giannis A Zacharas, Vasilis Ourailogloy, Eleni K Mouloudi, Eleni V Massa, Eva O Nagy, Electra E Stamou, Ellada V Kiourtzieva, Marina A Oikonomou, Luis E Avila, Cesar A Cortez, Johanna E Citalán, Sameer A Jog, Safal D Sable, Bhagyesh Shah, Mohan Gurjar, Arvind K Baronia, Mohammedfaruk Memon, Radhakrishnan Muthuchellappan, Venkatapura J Ramesh, Anitha Shenoy, Ramesh Unnikrishnan, Subhal B Dixit, Rachana V Rhayakar, Nagarajan Ramakrishnan, Vallish K Bhardwaj, Heera L Mahto, Sudha V Sagar, Vijayanand Palaniswamy, Deeban Ganesan, Seyed Mohammadreza Hashemian, Hamidreza Jamaati, Farshad Heidari, Edel A Meaney, Alistair Nichol, Karl M Knapman, Donall O’Croinin, Eimhin S Dunne, Dorothy M Breen, Kevin P Clarkson, Rola F Jaafar, Rory Dwyer, Fahd Amir, Olaitan O Ajetunmobi, Aogan C O’Muircheartaigh, Colin S Black, Nuala Treanor, Daniel V Collins, Wahid Altaf, Gianluca Zani, Maurizio Fusari, Savino Spadaro, Carlo A Volta, Romano Graziani, Barbara Brunettini, Salvatore Palmese, Paolo Formenti, Michele Umbrello, Andrea Lombardo, Elisabetta Pecci, Marco Botteri, Monica Savioli, Alessandro Protti, Alessia Mattei, Lorenzo Schiavoni, Andrea Tinnirello, Manuel Todeschini, Antonino Giarratano, Andrea Cortegiani, Sara Sher, Anna Rossi, Massimo M Antonelli, Luca M Montini, Paolo Casalena, Sergio Scafetti, Giovanna Panarello, Giovanna Occhipinti, Nicolò Patroniti, Matteo Pozzi, Roberto R Biscione, Michela M Poli, Ferdinando Raimondi, Daniela Albiero, Giulia Crapelli, Eduardo Beck, Vincenzo Pota, Vincenzo Schiavone, Alexandre Molin, Fabio Tarantino, Giacomo Monti, Elena Frati, Lucia Mirabella, Gilda Cinnella, Tommaso Fossali, Riccardo Colombo, Pierpaolo Terragni Ilaria Pattarino, Francesco Mojoli, Antonio Braschi, Erika E Borotto, Andrea N Cracchiolo, Daniela M Palma, Francesco Raponi, Giuseppe Foti, Ettore R Vascotto, Andrea Coppadoro, Luca Brazzi, Leda Floris, Giorgio A Iotti, Aaron Venti, Osamu Yamaguchi, Shunsuke Takagi, Hiroki N Maeyama, Eizo Watanabe, Yoshihiro Yamaji, Kazuyoshi Shimizu, Kyoko Shiozaki, Satoru Futami, Sekine Ryosuke, Koji Saito, Yoshinobu Kameyama, Keiko Ueno, Masayo Izawa, Nao Okuda, Hiroyuki Suzuki, Tomofumi Harasawa, Michitaka Nasu, Tadaaki Takada, Fumihito Ito, Shin Nunomiya, Kansuke Koyama, Toshikazu Abe, Kohkichi Andoh, Kohei Kusumoto, Akira Hirata, Akihiro Takaba, Hiroyasu Kimura, Shuhei Matsumoto, Ushio Higashijima, Hiroyuki Honda, Nobumasa Aoki, Hiroshi Imai, Yasuaki Ogino, Ichiko Mizuguchi, Kazuya Ichikado, Kenichi Nitta, Katsunori Mochizuki, Tomoaki Hashida, Hiroyuki Tanaka, Tomoyuki Nakamura, Daisuke Niimi, Takeshi Ueda, Yozo Kashiwa, Akinori Uchiyama, Olegs Sabelnikovs, Peteris Oss, Youssef Haddad, Kong Y Liew, Silvio A Ñamendys-Silva, Yves D Jarquin-Badiola, Luis A Sanchez-Hurtado, Saira S Gomez-Flores, Maria C Marin, Asisclo J Villagomez, Jordana S Lemus, Jonathan M Fierro, Mavy Ramirez Cervantes, Francisco Javier Flores Mejia, Dulce Dector, Dulce M Dector, Daniel R Gonzalez, Claudia R Estrella, Jorge R Sanchez-Medina, Alvaro Ramirez-Gutierrez, Fernando G George, Janet S Aguirre, Juan A Buensuseso, Manuel Poblano, Tarek Dendane, Amine Ali Zeggwagh, Hicham Balkhi, Mina Elkhayari, Nacer Samkaoui, Hanane Ezzouine, Abdellatif Benslama, Mourad Amor, Wajdi Maazouzi, Nedim Cimic, Oliver Beck, Monique M Bruns, Jeroen A Schouten, Myra Rinia, Monique Raaijmakers, Leo M Heunks, Hellen M Van Wezel, Serge J Heines, Ulrich Strauch, Marc P Buise, Fabienne D Simonis, Marcus J Schultz, Jennifer C Goodson, Troy S Browne, Leanlove Navarra, Anna Hunt, Robyn A Hutchison, Mathew B Bailey, Lynette Newby, Colin Mcarthur, Michael Kalkoff, Alex Mcleod, Jonathan Casement, Danielle J Hacking, Finn H Andersen, Merete S Dolva, Jon H Laake, Andreas Barratt-Due, Kim Andre L Noremark, Eldar Søreide, Brit Å Sjøbø, Anne B Guttormsen, Hector H Leon Yoshido, Ronald Zumaran Aguilar, Fredy A Montes Oscanoa, Alain U Alisasis, Joanne B Robles, Rossini Abbie B Pasanting-Lim, Beatriz C Tan, Pawel Andruszkiewicz, Karina Jakubowska, Cristina M Coxo, António M Alvarez, Bruno S Oliveira, Gustavo M Montanha, Nelson C Barros, Carlos S Pereira, António M Messias, Jorge M Monteiro, Ana M Araujo, Nuno T Catorze, Susan M Marum, Maria J Bouw, Rui M Gomes, Vania A Brito, Silvia Castro, Joana M Estilita, Filipa M Barros, Isabel M Serra, Aurelia M Martinho, Dana R Tomescu, Alexandra Marcu, Ovidiu H Bedreag, Marius Papurica, Dan E Corneci, Silvius Ioan Negoita, Evgeny Grigoriev, Alexey I Gritsan, Andrey A Gazenkampf, Ghaleb Almekhlafi, Mohamad M Albarrak, Ghanem M Mustafa, Khalid A Maghrabi, Nawal Salahuddin, Tharwat M Aisa, Ahmed S Al Jabbary, Edgardo Tabhan, Yaseen M Arabi, Yaseen M Arabi, Olivia A Trinidad, Hasan M Al Dorzi, Edgardo E Tabhan, Stefan Bolon, Oliver Smith, Jordi Mancebo, Hernan Aguirre-Bermeo, Juan C Lopez-Delgado, Francisco Esteve, Gemma Rialp, Catalina Forteza, Candelaria De Haro, Antonio Artigas, Guillermo M Albaiceta, Sara De Cima-Iglesias, Leticia Seoane-Quiroga, Alexandra Ceniceros-Barros, Antonio L Ruiz-Aguilar, Luis M Claraco-Vega, Juan Alfonso Soler, Maria del Carmen Lorente, Cecilia Hermosa, Federico Gordo, Miryam Prieto-González, Juan B López-Messa, Manuel P Perez, Cesar P Perez, Raquel Montoiro Allue, Ferran Roche-Campo, Marcos Ibañez-Santacruz, Susana Temprano, Maria C Pintado, Raul De Pablo, Pilar Ricart Aroa Gómez, Silvia Rodriguez Ruiz, Silvia Iglesias Moles, Mª Teresa Jurado, Alfons Arizmendi, Enrique A Piacentini, Nieves Franco, Teresa Honrubia, Meisy Perez Cheng, Elena Perez Losada, Javier Blanco, Luis J Yuste, Cecilia Carbayo-Gorriz, Francisca G Cazorla-Barranquero, Javier G Alonso, Rosa S Alda, Ángela Algaba, Gonzalo Navarro, Enrique Cereijo, Esther Diaz-Rodriguez, Diego Pastor Marcos, Laura Alvarez Montero, Luis Herrera Para, Roberto Jimenez Sanchez, Miguel Angel Blasco Navalpotro, Ricardo Diaz Abad, Raquel Montiel González, Dácil Parrilla Toribio, Alejandro G Castro, Maria Jose D Artiga, Oscar Penuelas, Tomas P Roser, Moreno F Olga, Elena Gallego Curto, Rocío Manzano Sánchez, Vallverdu P Imma, Garcia M Elisabet, Laura Claverias, Monica Magret, Ana M Pellicer, Lucia L Rodriguez, Jesús Sánchez-Ballesteros, Ángela González-Salamanca, Antonio G Jimenez, Francisco P Huerta, David D Llinares Moya, Alec A Tallet Alfonso, Palazon Sanchez Eugenio Luis, Palazon Sanchez Cesar, Sánchez I Rafael, Corcoles G Virgilio, Noelia N Recio, Richard O Adamsson, Christian C Rylander, Bernhard Holzgraefe, Lars M Broman, Joanna Wessbergh, Linnea Persson, Fredrik Schiöler, Hans Kedelv, Anna Oscarsson Tibblin, Henrik Appelberg, Lars Hedlund, Johan Helleberg, Karin E Eriksson, Rita Glietsch, Niklas Larsson, Ingela Nygren, Silvia L Nunes, Anna-Karin Morin, Thomas Kander, Anne Adolfsson, Lise Piquilloud, Hervé O Zender, Corinne Leemann-Refondini, Souheil Elatrous, Slaheddine Bouchoucha, Imed Chouchene, Islem Ouanes, Asma Ben Souissi, Salma Kamoun, Oktay Demirkiran, Mustafa Aker, Emre Erbabacan, Ilkay Ceylan, Nermin Kelebek Girgin, Menekse Ozcelik, Necmettin Ünal, Basak Ceyda Meco, Onat O Akyol, Suleyman S Derman, Barry Kennedy, Ken Parhar, Latha Srinivasa, Lia McNamee, Danny McAuley, Phil Hopkins, Clare Mellis, Vivek Kakar, Dan Hadfield, Andre Vercueil, Kaushik Bhowmick, Sally K Humphreys, Andrew Ferguson, Raymond Mckee, Ashok S Raj, Danielle A Fawkes, Philip Watt, Linda Twohey, Rajeev R JhaMatthew Thomas, Alex Morton, Varsha Kadaba, Mark J Smith, Anil P Hormis, Santhana G Kannan, Miriam Namih, Henrik Reschreiter, Julie Camsooksai, Alek Kumar, Szabolcs Rugonfalvi, Christopher Nutt, Orla Oneill, Colette Seasman, Ged Dempsey, Christopher J Scott, Helen E Ellis, Stuart Mckechnie, Paula J Hutton, Nora N Di Tomasso, Michela N Vitale, Ruth O Griffin, Michael N Dean, Julius H Cranshaw, Emma L Willett, Nicholas Ioannou, Sarah Gillis, Peter Csabi, Rosaleen Macfadyen, Heidi Dawson, Pieter D Preez, Alexandra J Williams, Owen Boyd, Laura Ortiz-Ruiz De Gordoa, Jon Bramall, Sophie Symmonds, Simon K Chau, Tim Wenham, Tamas Szakmany, Piroska Toth-Tarsoly, Katie H Mccalman, Peter Alexander, Lorraine Stephenson, Thomas Collyer, Rhiannon Chapman, Raphael Cooper, Russell M Allan, Malcolm Sim, David W Wrathall, Donald A Irvine, Kim S Zantua, John C Adams, Andrew J Burtenshaw, Gareth P Sellors, Ingeborg D Welters, Karen E Williams, Robert J Hessell, Matthew G Oldroyd, Ceri E Battle, Suresh Pillai, Istvan Kajtor, Mageswaran Sivashanmugavel, Sinead C Okane, Adrian Donnelly, Aniko D Frigyik, Jon P Careless, Martin M May, Richard Stewart, T John Trinder, Samantha J Hagan, Matt P Wise, Jade M Cole, Caroline C MacFie, Anna T Dowling, Javier Hurtado, Nicolás Nin, Javier Hurtado, Edgardo Nuñez, Gustavo Pittini, Ruben Rodriguez, María C Imperio, Cristina Santos, Ana G França, Alejandro EBEID, Alberto Deicas, Carolina Serra, Aditya Uppalapati, Ghassan Kamel, Valerie M Banner-Goodspeed, Jeremy R Beitler, Satyanarayana Reddy Mukkera, Shreedhar Kulkarni, Jarone Lee, Tomaz Mesar, John O Shinn Iii, Dina Gomaa, Christopher Tainter, Jarone Lee, Tomaz Mesar, Jarone Lee, Dale J Yeatts, Jessica Warren, Michael J Lanspa, Russel R Miller, Colin K Grissom, Samuel M Brown, Philippe R Bauer, Ryan J Gosselin, Barrett T Kitch, Jason E Cohen, Scott H Beegle, Renaud M Gueret, Aiman Tulaimat, Shazia Choudry, William Stigler, Hitesh Batra, Nidhi G Huff, Keith D Lamb, Trevor W Oetting, Nicholas M Mohr, Claine Judy, Shigeki Saito, Fayez M Kheir, Fayez Kheir, Adam B Schlichting, Angela Delsing, Daniel R Crouch, Mary Elmasri, Daniel R Crouch, Dina Ismail, Kyle R Dreyer, Thomas C Blakeman, Kyle R Dreyer, Dina Gomaa, Rebecca M Baron, Carolina Quintana Grijalba, Peter C Hou, Raghu Seethala, Imo Aisiku, Galen Henderson, Gyorgy Frendl, Sen-Kuang Hou, Robert L Owens, Ashley Schomer, Vesna Bumbasirevic, Bojan Jovanovic, Maja Surbatovic, Milic Veljovic, the ESICM Trials Group

**Affiliations:** 1 Centre for Experimental Medicine, Queen’s University Belfast, 97 Lisburn Road, Belfast, BT9 7BL Northern Ireland; 2 Regional Intensive Care Unit, Royal Victoria Hospital, 274 Grosvenor Road, Belfast, BT12 6BA Northern Ireland; 3 Intensive Care Unit, Royal Adelaide Hospital, North Terrace, Adelaide, SA 5000 Australia; 4 Research Centre on Public Health, School of Medicine and Surgery, University of Milan-Bicocca, Monza, Italy; 5 Discipline of Anaesthesia, School of Medicine, National University of Ireland, Galway, Ireland; 6 Departments of Anesthesia and Critical Care Medicine, St Michael’s Hospital, Toronto, Canada; 7 Keenan Research Centre for Biomedical Science, St Michael’s Hospital, Toronto, Canada; 8 Departments of Anesthesia and Physiology, University of Toronto, Toronto, Canada; 9 Interdepartmental Division of Critical Care Medicine, University of Toronto, Toronto, Canada; 10 School of Medicine and Surgery, University of Milan-Bicocca, Via Cadore 48, Monza, Italy; 11 Department of Emergency and Intensive Care, San Gerardo Hospital, Via Pergolesi 33, Monza, Italy; 12 Sorbonne Universités, UPMC Université Paris 06, Paris, France; 13 Istituto di Anestesia e Rianimazione, Università degli Studi di Milano, Ospedale Maggiore, Istituto di Ricovero e Cura a Carattere Scientifico, Milan, Italy; 14 Division of Pulmonary and Critical Care Unit, Department of Medicine, Massachusetts General Hospital, Harvard Medical School, Boston, USA; 15 Intensive Care Unit, The Royal Melbourne Hospital, The University of Melbourne, Melbourne, Australia

**Keywords:** Acute hypoxemic respiratory failure, Acute respiratory distress syndrome, Diabetes mellitus, LUNG SAFE

## Abstract

**Background:**

Diabetes mellitus is a common co-existing disease in the critically ill. Diabetes mellitus may reduce the risk of acute respiratory distress syndrome (ARDS), but data from previous studies are conflicting. The objective of this study was to evaluate associations between pre-existing diabetes mellitus and ARDS in critically ill patients with acute hypoxemic respiratory failure (AHRF).

**Methods:**

An ancillary analysis of a global, multi-centre prospective observational study (LUNG SAFE) was undertaken. LUNG SAFE evaluated all patients admitted to an intensive care unit (ICU) over a 4-week period, that required mechanical ventilation and met AHRF criteria. Patients who had their AHRF fully explained by cardiac failure were excluded. Important clinical characteristics were included in a stepwise selection approach (forward and backward selection combined with a significance level of 0.05) to identify a set of independent variables associated with having ARDS at any time, developing ARDS (defined as ARDS occurring after day 2 from meeting AHRF criteria) and with hospital mortality. Furthermore, propensity score analysis was undertaken to account for the differences in baseline characteristics between patients with and without diabetes mellitus, and the association between diabetes mellitus and outcomes of interest was assessed on matched samples.

**Results:**

Of the 4107 patients with AHRF included in this study, 3022 (73.6%) patients fulfilled ARDS criteria at admission or developed ARDS during their ICU stay. Diabetes mellitus was a pre-existing co-morbidity in 913 patients (22.2% of patients with AHRF). In multivariable analysis, there was no association between diabetes mellitus and having ARDS (OR 0.93 (0.78–1.11); *p* = 0.39), developing ARDS late (OR 0.79 (0.54–1.15); *p* = 0.22), or hospital mortality in patients with ARDS (1.15 (0.93–1.42); *p* = 0.19). In a matched sample of patients, there was no association between diabetes mellitus and outcomes of interest.

**Conclusions:**

In a large, global observational study of patients with AHRF, no association was found between diabetes mellitus and having ARDS, developing ARDS, or outcomes from ARDS.

**Trial registration:**

NCT02010073. Registered on 12 December 2013.

**Electronic supplementary material:**

The online version of this article (10.1186/s13054-018-2158-y) contains supplementary material, which is available to authorized users.

## Background

Acute hypoxemic respiratory failure (AHRF) is a common cause of admission to the intensive care unit (ICU). Many patients with AHRF will meet criteria for acute respiratory distress syndrome (ARDS), whilst those that do not meet the criteria remain at risk of developing it. ARDS remains a common condition in the critically ill and is associated with high mortality [1]. The long-term sequelae of ARDS are considerable, with substantive reductions in long-term quality of life [2, 3].

Despite numerous clinical trials there remain few therapeutic options for patients with ARDS [4]. Recent studies have identified subphenotypes of ARDS, which have a differential response to treatment [5]. The variety of pre-disposing conditions and inciting events for ARDS contributes to the heterogeneity of patients with this condition. It has been hypothesised that this heterogeneity may explain why therapies that were effective in exploratory studies were not shown to be effective in phase III trials [4]. Targeting interventions to prevent ARDS in patients at higher risk may prove more effective, and it is therefore necessary to identify those groups of patients who have a higher risk of ARDS.

Diabetes mellitus is common [6], with 4.4% of the world’s population anticipated to have a diagnosis by 2030 [7]. Observational data suggest that 40% of patients admitted to ICUs have a pre-existing diagnosis of diabetes mellitus [8]. Diabetes mellitus may protect against the development of ARDS. Whilst several studies indicated that patients with diabetes mellitus are less likely to develop ARDS [9–14], others did not demonstrate any protective effect of diabetes mellitus [15, 16]. The effect of diabetes mellitus on the risk of mortality in critically ill patients is also unclear, with most [17–19], but not all [16], data suggesting that diabetes mellitus is not associated with an increased risk of mortality. No studies have examined this relationship since the introduction of the current Berlin definition of ARDS [20]. Therefore, there remains a need to clarify and define any associations between diabetes mellitus and this syndrome. Any potential association could have significant impact on a clinician’s evaluation of a patient’s anticipated clinical outcome and may help to inform clinical trials evaluating therapies to prevent and treat ARDS.

The Large observational study to UNderstand the Global impact of Severe Acute respiratory FailurE (LUNG SAFE) undertaken in 459 ICUs in 50 countries [21] provides a unique opportunity to evaluate associations between pre-existing diabetes mellitus and the presence of ARDS in critically ill patients with acute hypoxemic respiratory failure. Secondary objectives of this analysis were to explore for any association of diabetes mellitus with the progression of AHRF to ARDS, and subsequent clinical outcome.

## Methods

### Study design

An ancillary analysis of LUNG SAFE was performed. This was a global, multi-centre prospective cohort study that enrolled 4499 patients with AHRF in 459 ICUs across 50 countries. The details of this study have previously been described [21]. Briefly, all patients admitted to a participating ICU were screened daily for AHRF (defined as the concurrent presence of (1) a ratio of partial pressure of arterial blood oxygen content to inspired fraction of oxygen (PaO_2_:FiO_2_ ratio) ≤ 300 mmHg, (2) acute pulmonary infiltrates identified on chest x-ray or computed tomography and (3) mechanical ventilation with a positive end-expiratory pressure (PEEP) of at least 5 cmH_2_O) during an enrolment period of 4 consecutive weeks in winter. Exclusion criteria were age below 16 years or inability to obtain informed consent (when required by local regulations).

Data were collected until day 28 (days 1, 2, 3, 5, 7, 14, 21 and 28), ICU discharge or death (whichever occurred earlier). Day 1 was defined as the day in which the patient met criteria for AHRF (baseline). Outcome data were recorded at hospital discharge or at day 90 (whichever occurred earliest). Meaurement of partial pressure of arterial carbon dioxide (PaCO_2_) was collected daily. Bicarbonate (${\mathrm{HCO}}_3^{-}$) data were not collected, instead the data were derived by the Henderson–Hasselbalch equation:

$$ {\mathrm{HCO}}_3^{-}=0.03\times {\mathrm{P}}_{\mathrm{a}}{\mathrm{CO}}^2\times {10}^{\mathrm{pH}-6.1}. $$

Using prospectively identified data, ARDS was defined according to the Berlin ARDS definition [20]: (1) presence of AHRF criteria, (2) onset within 1 week of insult or new or worsening respiratory symptoms, (3) bilateral opacities on chest x-ray or computed tomography and (4) cardiac failure not being the primary cause of AHRF. Patients were identified as having diabetes mellitus if it was a documented co-morbidity. The data collection form used in LUNG SAFE did not allow for inclusion of diabetes type, medications taken, or glycaemic control, and therefore this information was not available for this ancillary analysis. Furthermore, data were only collected for patients who met AHRF criteria. Each site investigator in LUNG SAFE was responsible for ensuring data integrity. No specific guidance on the diagnosis of cardiac failure was given to sites, and the method used to determine whether it was the sole explanation for AHRF was not recorded.

### Outcomes

The primary outcome was presence of ARDS. This was defined as meeting criteria for ARDS at any time during the follow-up period from meeting AHRF criteria. Secondary outcomes included development of ARDS (defined as occurring after day 2 from meeting AHRF criteria), duration of invasive mechanical ventilation, and hospital mortality (defined as outcome at day 90, or hospital discharge, whichever occurred earliest) in patients with ARDS. Duration of invasive mechanical ventilation was calculated as the number of days between the date of intubation and the date of extubation (or death, if the patient died during invasive mechanical ventilation).

### Statistical analysis

Descriptive statistics included calculation of proportions for categorical variables and mean (± standard deviation) for continuous variables. The AHRF population was stratified according to the presence of diabetes mellitus, and the statistical difference between groups (diabetic patients, non-diabetic patients) was evaluated by chi-square test (or Fisher exact test) for discrete variables and by *t* test or Wilcoxon rank sum test for continuous variables. The Shapiro–Wilks test was applied to assess normality of data distribution.

Patients were excluded from the primary analysis if cardiac failure was the only cause of AHRF. All clinical variables and covariates were entered into multivariable logistic regression model with variable selection based on a stepwise approach (forward and backward selection combined with a significance level of 0.05 both for entry and retention) to identify a set of independent variables associated with having ARDS at any time during follow up and after the second day after meeting AHRF criteria and with hospital mortality. A documented co-morbidity of diabetes mellitus (“Diabetes diagnosis”) was locked into the multivariable model as it was the primary exposure of interest. As most patients in LUNG SAFE met ARDS criteria within 48 h of AHRF onset [21], those patients who met ARDS criteria after 48 h were studied separately, and were deemed to have developed ARDS. If diabetes mellitus was not detected as a statistically significant predictor in the final multivariable logistic regression model, for each outcome the entry of this variable was forced into the model. We also evaluated in the models the interaction term “diabetes and presence of a pulmonary ARDS risk factor” in order to assess a possible different effect of diabetes mellitus on outcomes in patients with or without a pulmonary ARDS risk factor. Results of logistic models are shown as odds ratios (ORs) with 95% confidence intervals (CIs) and *p* value. No assumptions were made for missing data.

To account for the differences in baseline characteristics between patients with AHRF, propensity score was used to match (1:1 without replacement) diabetic and non-diabetic patients. Propensity score was estimated using a logistic regression model that had “presence of diabetes” as the response variable and that contained as predictors sex, age, body mass index (BMI), non-pulmonary Sequential Organ Failure Assessment (SOFA) score (adjusted for missing values), co-morbidities (chronic liver failure, chronic renal failure, chronic heart failure, haematologic neoplasm, immunosuppression, active neoplasm, chronic obstructive pulmonary disease (COPD) or home ventilation), ARDS risk factors and ventilator variables at baseline. The balance in measured variables between groups (diabetic and non-diabetic subjects) has been assessed using standardised difference and a value of less than 0.10 likely denoted a negligible imbalance. The association between diabetes mellitus and outcomes of interest was assessed in matched samples using McNemar’s test for dichotomous outcomes and Wilcoxon signed ranks test for continuous outcomes.

Kaplan–Meier analysis was applied to estimate the probability of hospital mortality within 90 days of AHRF onset. It was assumed that patients discharged alive from hospital before 90 days were alive at day 90. The difference in survival curves between diabetic and non-diabetic patients was evaluated using the log-rank test.

All *p* values were two-sided, and a *p* value less than 0.05 was considered statistically significant. Statistical analyses were performed using SAS software, version 9.4 (SAS Institute, Cary, NC, USA) and with R, version 3.3.3. (R Project for Statistical Computing, https://www.r-project.org/).

## Results

AHRF was identified in 4499 patients: 392 patients (8.7%) were excluded from the primary analysis because their respiratory failure was fully explained by cardiac failure (Additional file 1: Figure S1). Of the remaining 4107 patients with AHRF, 3022 patients (73.6%) fulfilled ARDS criteria within 2 days of AHRF onset (*N* = 2813; 93.1%) or developed ARDS during their ICU admission (*N* = 209; 6.9%). A total of 913 patients (22.2%) had diabetes mellitus and of these, 657 (72.0%) met ARDS criteria during their ICU admission (Additional file 1: Table S1). The baseline characteristics of patients with AHRF are summarised in Table 1. Patients with diabetes mellitus were older, had a higher BMI, received mechanical ventilation with larger tidal volumes and higher plateau pressures, had more co-morbidities but were less likely to have a risk factor for ARDS. Active neoplasm, haematological neoplasm and immunosuppression occurred more frequently in non-diabetic patients.

**Table 1:** Baseline characteristics of patients with AHRF (stratified by presence of diabetes)

	Overall (*N* = 4107)	Diabetic (*N* = 913)	Non-diabetic (*N* = 3194)	*p* value (Diabetic vs. non-diabetic)
Age (years)	61.7 ± 16.7	66.6 ± 13.1	60.3 ± 17.4	< 0.0001
Male – *N* (%)	2547 (62.0)	575 (63.0)	1972 (61.7)	0.50
BMI (kg/m^2^)^a^	27.5 ± 8.3	30.4 ± 11.7	26.6 ± 6.9	< 0.0001
Non-pulmonary SOFA score (adjusted for missing values)^b^	6.1 ± 4.1	6.2 ± 4.2	6.1 ± 4.0	0.39
ARDS risk factors – *N* (%)				0.07
Pulmonary	2295 (55.9)	511 (52.0)	1784 (55.9)	
Non-pulmonary	833 (20.3)	174 (19.1)	659 (20.6)	
Pulmonary and non-pulmonary	559 (13.6)	115 (12.6)	444 (13.9)	
No risk factor	420 (10.2)	113 (12.4)	307 (9.6)	
Co-morbidities – *N* (%)				
No co-morbidity other than diabetes mellitus	1958 (47.7)	343 (37.6)	1615 (50.6)	< 0.0001
Chronic liver failure	161 (3.9)	33 (3.6)	128 (4.0)	0.37
Chronic renal failure	428 (10.4)	205 (22.5)	227 (7.0)	< 0.0001
Chronic cardiac failure (NYHA III-IV)	428 (10.4)	156 (17.1)	272 (8.5)	< 0.0001
COPD or home ventilation	989 (24.1)	294 (32.2)	695 (21.8)	< 0.0001
Active neoplasm	368 (9.0)	61 (6.7)	307 (9.6)	0.006
Immunosuppression	453 (11.0)	73 (8.0)	380 (11.9)	0.0009
Haematologic neoplasm	170 (4.1)	22 (2.4)	148 (4.6)	0.003
Active neoplasm/immunosuppression/haematologic neoplasm	820 (20.0)	134 (14.7)	686 (21.5)	< 0.0001
Baseline ventilator variables				
PaO_2_:FiO_2_ ratio (mmHg)^c^	167.5 ± 67.8	169.2 ± 67.1	167.0 ± 68.0	0.35
Tidal volume (ml/kg PBW)^d^	7.8 ± 2.2	8.0 ± 2.4	7.7 ± 2.1	0.05
Plateau pressure (cmH_2_O)^e^	22.4 ± 5.9	23.3 ± 5.6	22.1 ± 6.0	0.001
PEEP (cmH_2_O)^f^	7.8 ± 3.1	7.8 ± 3.1	7.8 ± 3.1	0.76
Peak inspiratory pressure (cmH_2_O)^g^	24.8 ± 8.7	25.3 ± 8.8	24.7 ± 8.7	0.05
Respiratory rate (breaths/min)^h^	21.2 ± 8.1	20.9 ± 6.7	21.3 ± 8.4	0.42
Blood pH^i^	7.34 ± 0.12	7.33 ± 0.13	7.34 ± 0.12	0.06
PaCO_2_ (mmHg)^j^	45.8 ± 15.6	46.0 ± 15.6	45.7 ± 15.6	0.51
Bicarbonate (mmol/L)^k^	23.6 ± 6.8	23.4 ± 7.1	23.6 ± 6.7	0.36

Data presented as mean ± standard deviation unless otherwise stated

*Abbreviations: AHRF* acute hypoxaemic respiratory failure *ARDS* acute respiratory distress syndrome, *BMI* body mass index, *COPD* chronic obstructive pulmonary disease, *NYHA* New York heart association functional classification, *PaCO*_*2*_ partial pressure of carbon dioxide in arterial blood, *PBW* predicted body weight, *PEEP* positive end-expiratory pressure, *SOFA* sequential organ failure assessment

^a^872 diabetic, 3010 non-diabetic, 3882 patients overall

^b^908 diabetic, 3168 non-diabetic, 4076 patients overall

^c^911 diabetic, 3175 non-diabetic, 4086 patients overall

^d^830 diabetic, 2840 non-diabetic, 3670 patients overall

^e^244 diabetic, 760 non-diabetic, 1004 patients overall

^f^910 diabetic, 3163 non-diabetic, 4073 patients overall

^g^864 diabetic, 2985 non-diabetic, 3849 patients overall

^h^909 diabetic, 3159 non-diabetic, 4068 patients overall

^i^896 diabetic, 3146 non-diabetic, 4042 patients overall

^j^894 diabetic, 3146 non-diabetic, 4040 patients overall

^k^894 diabetic, 3145 non-diabetic, 4039 patients overall

### Presence of ARDS (at any time)

There was no difference in the incidence of ARDS between patients with and without diabetes mellitus (72% with vs. 74% without diabetes mellitus; *p* = 0.21) (Additional file 1: Table S1). In multivariable analysis, there was no association between diabetes mellitus and having ARDS (OR 0.93 (0.78–1.11); *p* = 0.39). Haematological neoplasm, baseline PaO_2_:FiO_2_ ratio, respiratory rate, PEEP and peak inspiratory pressure were significantly associated with having ARDS, whilst COPD or home ventilation were associated with a reduced likelihood of having ARDS (Table 2). No difference in the relationship between diabetes and incidence of ARDS was detected between patients with or without a pulmonary risk factor (*p* = 0.99, data not shown).

**Table 2:** Multivariable analysis of factors associated with having ARDS

Variable	Odds ratio (95% CI)	*p* value
Diabetes mellitus diagnosis	0.93 (0.78, 1.11)	0.39
Baseline PaO_2_:FiO_2_ ratio (mmHg)	0.996 (0.994, 0.997)	< 0.0001
Baseline respiratory rate (breaths/min)	1.03 (1.02, 1.04)	< 0.0001
Baseline PEEP (cmH_2_O)	1.06 (1.04, 1.11)	< 0.0001
Baseline peak inspiratory pressure (cmH_2_O)	1.02 (1.01, 1.03)	0.0001
ARDS risk factors^a^		
Pulmonary risk factors	1.93 (1.53, 2.45)	< 0.0001
Non-pulmonary risk factors	1.50 (1.15, 1.95)	0.003
Pulmonary and non-pulmonary risk factors	1.89 (1.40, 2.53)	< 0.0001
COPD or home ventilation	0.78 (0.65, 0.93)	0.005
Haematologic neoplasm	1.60 (1.02, 2.52)	0.04

Analysis based on data from 3814 observations

*Abbreviations: ARDS* acute respiratory distress syndrome, *CI* confidence interval, *COPD* chronic obstructive pulmonary disease, *PEEP* positive end-expiratory pressure

^a^To estimate the odds ratio, the reference category is “No risk factor”

### Development of ARDS (after day 2)

A total of 209 patients developed ARDS after 2 days from meeting AHRF criteria. The proportion of patients who developed ARDS after day 2 was similar between the diabetic and non-diabetic cohort (4.8% vs. 5.2%; *p* = 0.67) (Additional file 1: Table S1).

Patients with diabetes mellitus who developed ARDS after day 2 were older and had more frequent co-existing chronic renal failure, than non-diabetic patients (Additional file 1: Table S2).

In patients who remained at risk of developing ARDS after day 2 from meeting AHRF criteria (*N* = 1294), there was no association identified in multivariable analysis between diabetes mellitus and developing ARDS (OR 0.79 (0.54–1.15); *p* = 0.22). Baseline peak inspiratory pressure, age, pulmonary risk factors and the combination of pulmonary and non-pulmonary risk factors were all associated with increased likelihood of developing ARDS after day 2 from meeting AHRF criteria (Table 3).

**Table 3:** Multivariable analysis for developing ARDS after day 2

Variable	Odds-ratio (95% CI)	*p* value
Diabetes mellitus diagnosis	0.79 (0.54, 1.15)	0.22
Age (years)	1.01 (1.00, 1.02)	0.03
ARDS risk factors^a^		
Pulmonary risk factors	2.03 (1.16, 3.54)	0.01
Non-pulmonary risk factors	1.66 (0.90, 3.08)	0.11
Pulmonary and non-pulmonary risk factors	2.32 (1.20, 4.48)	0.01
Baseline peak inspiratory pressure (cmH_2_O)	1.03 (1.01, 1.05)	0.007

Analysis based on data from 1194 observations

*Abbreviations: ARDS* acute respiratory distress syndrome, *CI* confidence interval

^a^To estimate odds ratio, the reference category is “No risk factor”

### Outcomes in ARDS

**Fig. 1:**
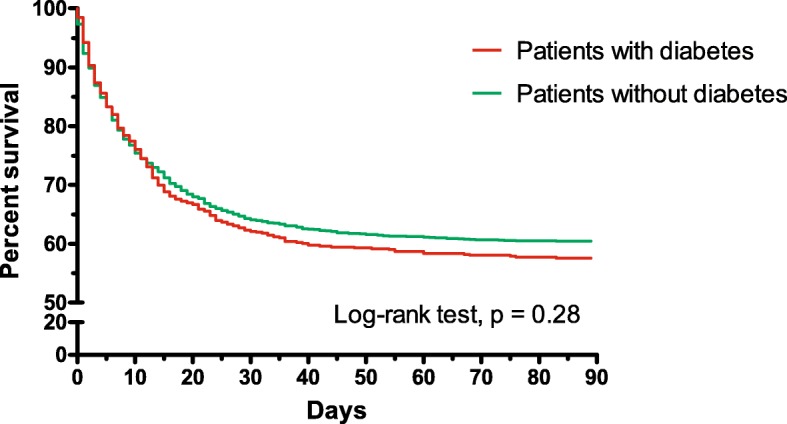
Hospital survival in diabetic and non-diabetic patients with acute respiratory distress syndrome (ARDS). Kaplan–Meier graph shows survival status for patients with and without diabetes mellitus. There was no difference in survival probability between the two groups (log-rank test, *p* = 0.28)

A total of 3022 patients had ARDS at any time during the follow-up period (Additional file 1: Table S1). Patients with diabetes mellitus who developed ARDS were older, had a higher BMI, received mechanical ventilation at baseline with higher tidal volumes and plateau pressure, and had more co-morbidities than non-diabetic patients. Documented immunosuppression (unrelated to diabetes mellitus) was more prevalent in those without diabetes mellitus (Additional file 1: Table S6). The duration of invasive mechanical ventilation was similar between the two groups overall (13.4 ± 17.0 days vs. 12.5 ± 13.4 days; *p* = 0.70) and in survivors and non-survivors. Hospital mortality was similar between the two groups (41.6% with vs. 38.8% without diabetes mellitus; *p* = 0.19) (Additional file 1: Table S7). There was no difference in survival probability between patients with and without diabetes mellitus (log-rank test, *p* = 0.28) (Fig. 1).

There was no association between diabetes mellitus and hospital mortality in those that had ARDS at any time when analysed using a univariate (Additional file 1: Table S8) or multivariate approach (OR 1.15 (0.93–1.42); *p* = 0.19) (Table 4). No difference in the relationship between diabetes mellitus and hospital mortality was detected between patients with or without a pulmonary ARDS risk factor (*p* = 0.26, data not shown). In patients who developed ARDS after day 2, there was no association between diabetes mellitus and hospital mortality (OR 1.07 (0.53–2.17); *p* = 0.84) (Additional file 1: Table S10).

**Table 4:** Multivariable analysis for hospital mortality in patients with ARDS

Variable	Odds ratio (95% CI)	*p* value
Diabetes mellitus diagnosis	1.15 (0.93, 1.42)	0.19
Age (years)	1.02 (1.02, 1.03)	< 0.0001
Non-respiratory SOFA (adjusted for missing values)	1.12 (1.10, 1.49)	< 0.0001
BMI (kg/m^2^)	0.98 (0.96, 0.99)	< 0.0001
Chronic cardiac failure (NYHA III-IV)	1.36 (1.04, 1.79)	0.03
Haematologic neoplasm	4.05 (2.59, 6.36)	< 0.0001
Active neoplasm	1.91 (1.43, 2.56)	< 0.0001
Immunosuppression	1.56 (1.19, 2.05)	0.001
Respiratory rate (breaths/min)	1.03 (1.02, 1.04)	< 0.0001
Baseline PaO_2_:FiO_2_ ratio (mmHg)	0.998 (0.997, 0.999)	0.007
Baseline PEEP (cmH_2_O)	0.95 (0.93, 0.98)	0.003
Baseline peak inspiratory pressure (cmH_2_O)	1.01 (1.00, 1.02)	0.05
Blood pH	0.18 (0.09, 0.39)	< 0.0001

Analysis based on data from 2633 observations

*Abbreviations: BMI* body mass index, *CI* confidence interval, *SOFA* Sequential Organ Failure Assessment, *NYHA* New York heart association functional classification, *PaO*_*2*_*:FiO*_*2*_ ratio of partial pressure of arterial blood oxygen content to inspired fraction of oxygen, *PEEP* positive end-expiratory pressure

### Assessing the impact of diabetes mellitus using propensity score matching

In a matched sample of patients with and without diabetes mellitus there was no difference in baseline characteristics (Additional file 1: Table S11). In this cohort of patients, diabetes mellitus was not associated with having ARDS (72.0% with vs. 72.8% without diabetes mellitus; *p* = 0.77), nor with developing ARDS after day 2 (5.2% vs. 5.7%; *p* = 0.52), nor with hospital mortality (40.3% vs. 38.3%; *p* = 0.48) (Additional file 1: Table S12).

### Presence of diabetes mellitus and respiratory muscle dysfunction

Additional file 1: Figure S2, shows blood PaCO_2_ (panel A) and bicarbonate (panel B) over time in diabetic and non-diabetic patients with AHRF. At day 5 after meeting AHRF criteria, patients with diabetes mellitus had a higher blood bicarbonate level, but no difference in blood PaCO_2_. In contrast, at day 14, patients with diabetes mellitus had a higher PaCO_2_ but did not have any difference in blood bicarbonate, compared to patients without diabetes mellitus.

## Discussion

In this large, global, multi-centre prospective observational study of patients with AHRF, pre-existing diabetes mellitus was not associated with the presence of ARDS in patients who have AHRF not fully explained by cardiac failure. In patients who remained at risk of developing ARDS after 48 h from having AHRF, there was no association between diabetes mellitus and developing ARDS. Finally, in both cohorts, diabetes mellitus did not modify outcomes.

Previous studies have evaluated whether there is an association between diabetes mellitus and risk of ARDS [9–16, 22], although the results are inconsistent. The LUNG SAFE cohort includes all patients meeting ARDS criteria and provide more robust data than prior observational studies because of the frequency of clinician under-recognition of ARDS [21]. Moreover, that LUNG SAFE comprises 3022 patients with ARDS from 459 ICUs in 50 countries means the findings are more generalizable. Although there are fewer patients with diabetes mellitus, this cohort included many more patients with ARDS than were included in a meta-analysis of this topic [23]. The findings presented in this study are relevant to clinicians evaluating the risk of a patient with a pre-existing history of diabetes mellitus developing ARDS in the setting of AHRF, as well as their outcome.

The limitations of this study include those inherent in observational studies. Epidemiological studies suggest that 5–15% of patients admitted to the ICU have unrecognised diabetes mellitus [8]. Given that data were not collected during LUNG SAFE to identify patients with unrecognised diabetes mellitus, a proportion of patients will be incorrectly categorised. Furthermore, this was not a pre-specified analysis in the LUNG SAFE study, and therefore does not include all the variables that would be of interest when investigating diabetes mellitus and ARDS. As a consequence, it is only possible to adjust for confounding factors that were collected as part of LUNG SAFE [21]. Like most of the previously published data, this cohort was not separated into different types of diabetes mellitus. Although patients with diabetes mellitus had a significantly higher BMI compared with non-diabetic patients (Table 1), we are unable to confirm if this reflects the majority of patients with diabetes mellitus being diagnosed as having type 2 diabetes mellitus. This is potentially important because of the different pathophysiological processes between the various forms of diabetes mellitus that may exert different effects in ARDS. However, in an exploratory analysis of a previous observational study, it was identified that both type 1 and type 2 diabetes mellitus were independently associated with reduced development of ARDS [14]. The results presented in this study are hypothesis generating. Any risk or protective effect associated with diabetes mellitus may be modified by the magnitude of pre-existing glucose intolerance, chronic end-organ complications of diabetes mellitus and chronic glucose-lowering drugs [24, 25]. Accordingly, future investigations of the relationship between diabetes mellitus and ARDS should aim to have a more rigorous screening process for diabetes mellitus and should consider evaluating an association between different types of diabetes mellitus and ARDS, as well as the confounding effects of treatment.

In LUNG SAFE, the criteria for AHRF determined that patients who were included in the analysis had significant lung injury at the point of entry into the study. Whilst this describes a significant proportion of patients who develop ARDS, the cohort of patients “at risk” of ARDS include those with less significant physiological derangement (e.g. patients with pneumonia but a PaO_2_:FiO_2_ ratio > 300 mmHg), and therefore does not include all patients at risk of ARDS. The LUNG SAFE study did not collect detailed data on all screened patients (Additional file 1: Figure S1). Therefore, it was not possible to assess the relationship between diabetes mellitus and ARDS in all patients at risk. This includes patients with respiratory failure who were not receiving ventilatory support, both within and outside the ICU environment, therefore limiting the applicability of these findings. Finally, it is possible that the sample of patients developing ARDS after day 2 was too small to detect an association with diabetes mellitus.

In this analysis of the LUNG SAFE cohort, patients who had AHRF only due to cardiac failure were excluded, providing a clearer description of the relationship between diabetes mellitus, ARDS and outcomes of AHRF. When these patients were included in the analysis, diabetes mellitus was associated with a reduced risk of developing ARDS (data not shown). This is explained by more patients with diabetes mellitus having their AHRF fully explained by cardiac failure (11.6% with vs. 7.8% without diabetes mellitus; *p* = 0.0002). Most prior observational studies that demonstrated a protective effect of diabetes mellitus on ARDS, did not exclude patients who developed cardiac failure from their at-risk population [9, 10, 12, 14]. In one study that excluded patients with cardiogenic pulmonary oedema at the onset of septic shock, patients with diabetes mellitus developed ARDS less frequently than those patients without diabetes mellitus; however, in a final multiple logistic regression analysis diabetes mellitus was not significantly associated with ARDS [16]. This demonstrates that combining patients who develop cardiac failure with those that have neither ARDS nor cardiac failure can cause a bias in the estimates of the effect of diabetes mellitus on ARDS [16], and the results presented in this analysis of LUNG SAFE support that finding. Future studies evaluating exposure risk in ARDS should consider excluding patients who develop acute hypoxaemic respiratory failure due to cardiac failure in their risk analysis.

Most patients met ARDS criteria within the first 48 h of having AHRF. In those patients that developed ARDS (i.e. after day two from AHRF), there was no association between diabetes mellitus and ARDS. This expands on the understanding gathered from previous observational studies. Patients with diabetes mellitus received more injurious mechanical ventilation at baseline (Table 1), and it is possible that ventilator-induced lung injury negated any protective effect that diabetes mellitus has upon the development of ARDS. There was no statistically significant association between diabetes mellitus and reduction in mortality in those who developed ARDS, a finding that supports prior observational data [9, 10, 12, 14].

The results of most previous studies have demonstrated that diabetes mellitus is not associated with increased ICU mortality [17–19]. However, in some circumstances diabetes mellitus may have a negative effect upon patients’ health and outcome from disease. For example, during the 2009 H1N1 influenza pandemic, diabetes mellitus was associated with more severe infection and with a greater risk of death [26]. Similarly, diabetes mellitus is associated with an increased risk of hospitalisation from community-acquired pneumonia and bacterial infection [27], with patients < 40 years of age at the highest risk when compared with age-matched controls [28]. ARDS can be sub-divided into “direct” and “indirect” based on the underlying insult, and both influenza and community-acquired pneumonia would be considered direct insults. Given the association between diabetes mellitus and more severe disease, it is plausible that there is a harmful association between diabetes mellitus and ARDS in patients who have direct risk factors. However, previous data have demonstrated that diabetes mellitus is independently associated with reduced risk of mortality in direct ARDS (defined as ARDS associated with gastric aspiration or pneumonia) [29]. Interestingly, in this ancillary analysis of LUNG SAFE, there was no association between diabetes mellitus and ARDS or hospital mortality among patients who had at least one pulmonary risk factor. This difference may be explained by the difference between the included patients, as LUNG SAFE included inhalational injury, pulmonary contusion, pulmonary vasculitis and drowning, alongside gastric aspiration and pneumonia, as direct risk factors.

Diabetes mellitus may act as a confounding factor for receiving insulin therapy, and it may be that the previously observed protective effects of diabetes mellitus in ARDS may reflect an immune-modulatory effect of medications such as insulin [30]. Pre-clinical studies have demonstrated protective benefits of insulin therapy in lung injury secondary to trauma [31], and when used to maintain euglycaemia in ARDS secondary to endotoxaemia [32]. However, other data suggest that immune hypo-responsiveness in diabetes mellitus is reversed by insulin therapy, and associated with alveolar neutrophil infiltration and increased alveolar concentration of pro-inflammatory cytokines in lipopolysaccharide (LPS)-induced lung injury [33], suggesting that insulin may restore immune function and therefore could reverse some of the protective effects of diabetes mellitus in relation to ARDS. Other medications that have been identified as having potential immuno-modulatory effects, including metformin [34, 35], aspirin [36] ace-inhibitors [4] and statins [37] may act as confounding factors in this study. Diabetic polyneuropathy has been demonstrated to affect respiratory neuromuscular function in patients with type 2 diabetes mellitus [38], but it is unknown whether diabetic polyneuropathy has a significant impact in patients with AHRF. In an assessment of blood PaCO_2_ and bicarbonate over time in patients with and without diabetes mellitus, there was no trend identified to suggest that respiratory muscle insufficiency was greater in either cohort. However, this analysis is limited, and further assessment of this important, potential confounding factor, may be warranted.

Patients with diabetes mellitus had significant differences in their baseline co-morbidities when compared to patients without diabetes mellitus. To account for this, a post-hoc propensity score was used to match patients with and without diabetes mellitus. This analysis did not demonstrate an association between diabetes mellitus and outcomes of interest, suggesting that the absence of an effect of diabetes mellitus in the wider AHRF population is unlikely to have been due to baseline population imbalance.

## Conclusions

In this global, multi-centre, prospective observational study of mechanically ventilated patients with AHRF, there was no association identified between diabetes mellitus and ARDS or outcomes. These findings are hypothesis generating and may inform clinicians about the risk of developing ARDS and the outcomes of patients with diabetes mellitus and AHRF. Further research is required to establish if there is an effect between the type and severity of diabetes mellitus, as well as the confounding effect of treatment for this condition, and the development and outcome of ARDS.

## Additional file

Additional file 1: Supplementary analysis, figures and tables. Contains supplementary results and data from this analysis of the LUNG SAFE database. Included are results relating to outcomes from patients who developed ARDS after day 2, a flowchart describing the study population, and tables supplementary to the results presented in the main manuscript. (DOCX 832 kb)

## Abbreviations

AHRF: Acute hypoxemic respiratory failure
ARDS: Acute respiratory distress syndrome
BMI: Body mass index
CI: Confidence interval
COPD: Chronic obstructive pulmonary disease
ICU: Intensive care unit
LUNG SAFE: Large observational study to understand the global impact of severe acute respiratory failure
NYHA: New York Heart Association
OR: Odds ratio
PaCO_2_: Partial pressure of arterial carbon dioxide
PaO_2_:FiO_2_ ratio: Ratio of partial pressure of arterial blood oxygen content to inspired fraction of oxygen
PEEP: Positive end-expiratory pressure
SOFA: Sequential Organ Failure Assessment
