# Supplementary material for: Identifying associations between diabetes and acute respiratory distress syndrome in patients with acute hypoxemic respiratory failure: an analysis of the LUNG SAFE database
Source: Crit Care. 2018 Oct 27;22:268. doi: 10.1186/s13054-018-2158-y (PMC6203969; doi:10.1186/s13054-018-2158-y)
Supplement: Supplementary file 1 — Supplementary analysis, figures and tables. Contains supplementary results and data from this analysis of the LUNG SAFE database. Included are results relating to outcomes from patients who developed ARDS after day 2, a flowchart describing the study population, and tables supplementary to the results presented in the main manuscript. (DOCX 832 kb) [file 13054_2018_2158_MOESM1_ESM.docx]

**Additional file 1**

*Outcomes in patients who develop ARDS after day 2*

In patients who developed ARDS after day 2 (Table S2), there was no difference in duration of invasive mechanical ventilation between patients with and without diabetes (14 (8 – 21) vs. 13 (8 – 20); p = 0.95) (Table S4). Hospital mortality was similar between at-risk patients with and without diabetes mellitus (40.9% vs. 36.4%; p = 0.58) (Table S4). In multivariate analysis only increasing age (1.03 [1.01 – 1.05]; p = 0.008) and immunosuppression (3.89 [1.11 – 13.61]; p = 0.03) were associated with increased hospital mortality from ARDS (Table S10).

**Figure S1: Flowchart of study population**

**Figure S2: Blood gas variables patients with AHRF stratified to presence of diabetes**


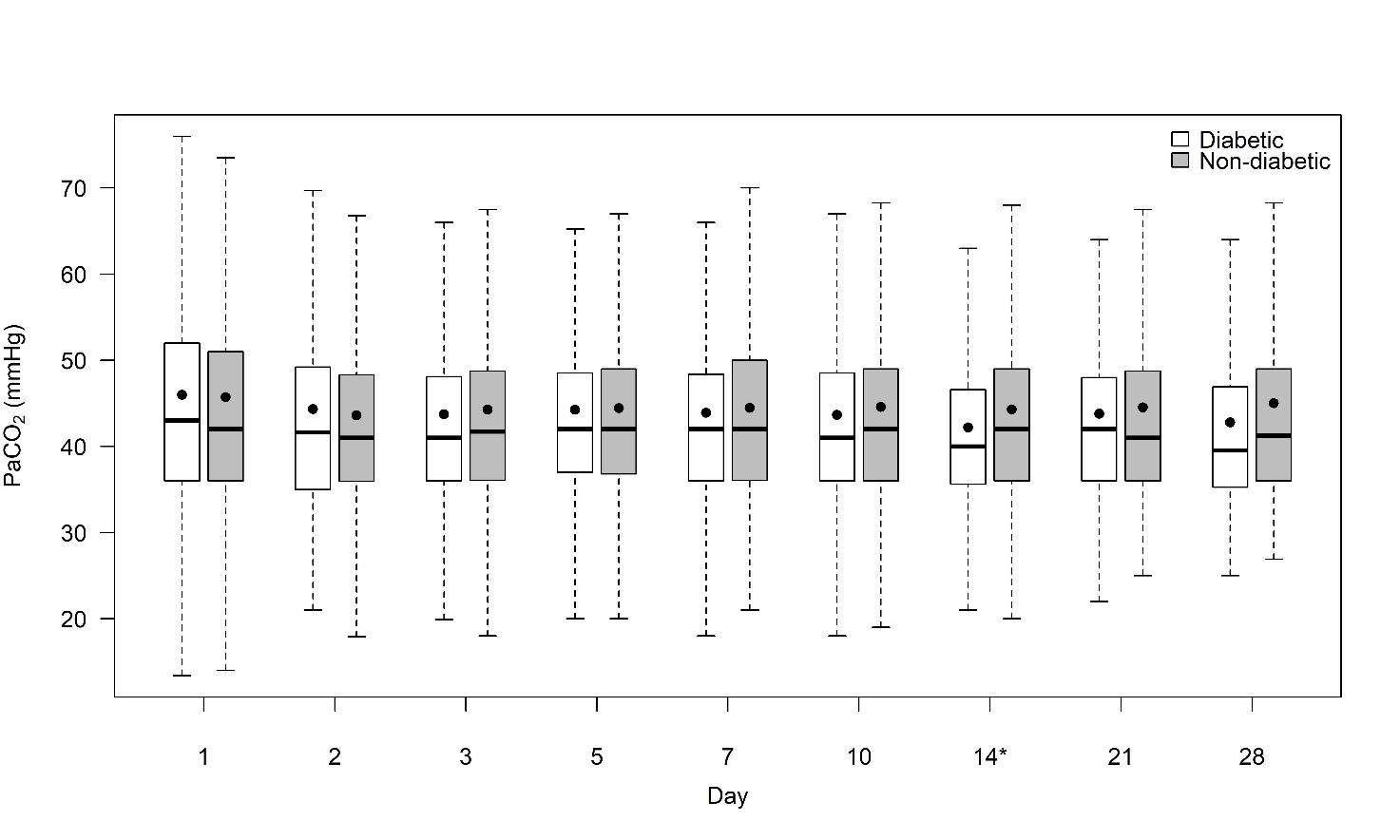

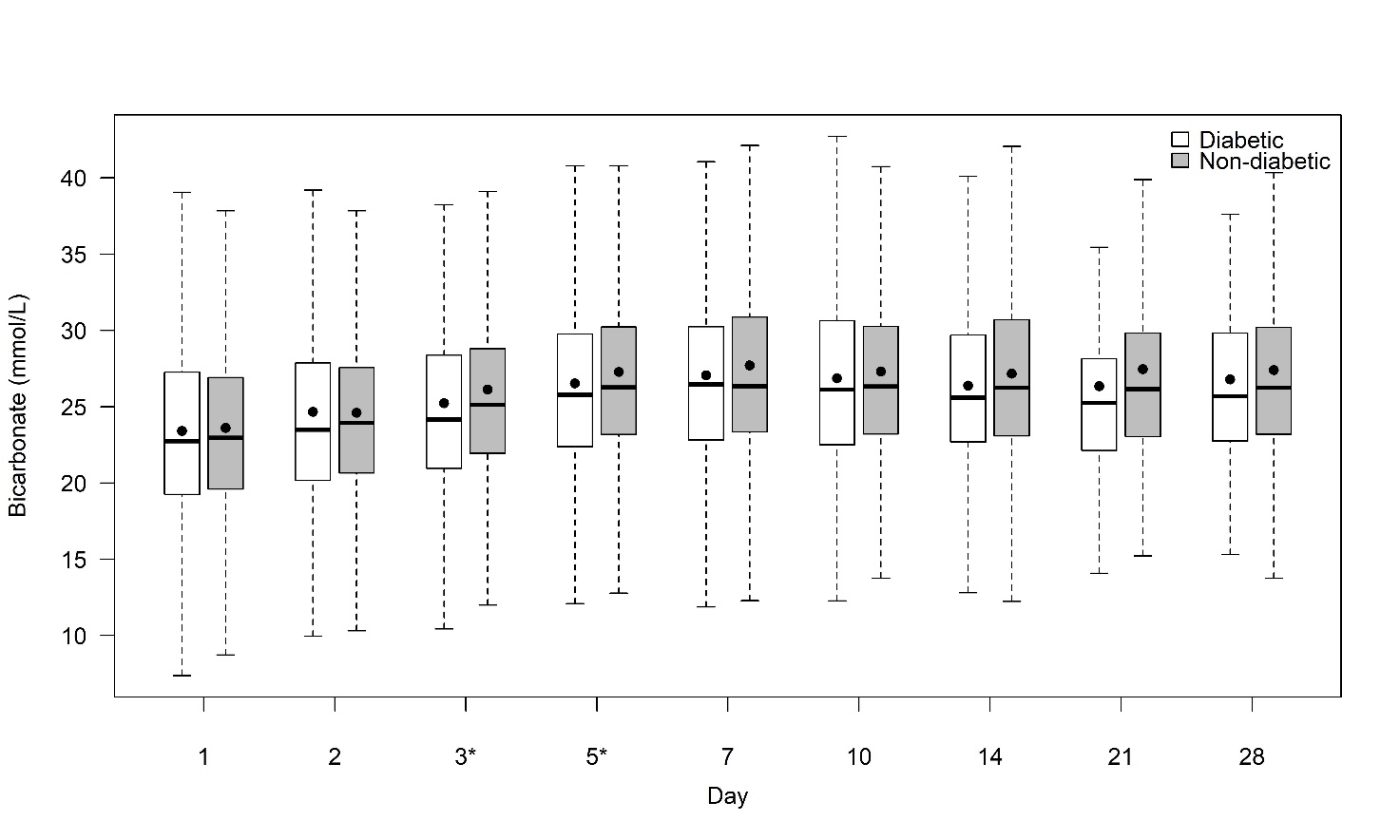


**A**

**B**

Figure S2: A: PaCO_2_, and B. Bicarbonate, during follow-up period in AHRF patients, stratified according to presence of diabetes. * denotes p-value < 0.05 for difference between diabetic and non-diabetic.

**Table S1: Outcomes from AHRF**

| **Outcome** | **Overall**  (N = 4107) | **Diabetic**  (N = 913) | **Non-diabetic**  (N = 3194) | **p-value** |
| --- | --- | --- | --- | --- |
| Duration of invasive mechanical ventilation (days)^a^ | 8 (4 – 14) | 7 (4 – 14) | 8 (4 – 15) | 0.89 |
| Duration of invasive mechanical ventilation in survivors at hospital discharge (days)^b^ | 8 (4 – 14) | 7 (4 – 13.5) | 8 (4 – 14) | 0.67 |
| Duration of invasive mechanical ventilation in non-survivors at hospital discharge (days)^c^ | 8 (3 – 15) | 8 (4 – 14) | 7 (3 – 15) | 0.52 |
| Having ARDS ever – N (%) | 3022 (73.6) | 657 (72.0) | 2365 (74.0) | 0.21 |
| Developed ARDS late (after day 2) – N (%) | 209 (6.9) | 44 (4.8) | 165 (5.2) | 0.67 |
| Time to ARDS from meeting AHRF criteria (days)^d^ | 0 (0 – 0) | 0 (0 – 0) | 0 (0 – 0) | 0.46 |
| Hospital mortality – N (%)^e^ | 1531 (37.4) | 355 (39.1) | 1176 (36.9) | 0.24 |

Data presented as median (interquartile range) unless otherwise stated.

Abbreviations: AHRF: acute hypoxaemic respiratory failure; ARDS: acute respiratory distress syndrome.

A: 759 diabetic, 2704 non-diabetics, 3463 overall; B: 432 diabetic, 1610 non-diabetic, 2042 overall; C: 323 diabetic, 1086 non-diabetic, 1409 overall; D: 657 diabetics, 2365 non-diabetics, 3022 overall; E: 908 diabetics, 3183 non-diabetics, 4091 overall

**Table S2: Baseline characteristics of patients who developed ARDS after day 2**

| **Variable** | **Overall**  (n = 209) | **Diabetics**  (n = 44; 21.1%) | **Non-diabetics**  (n = 165; 78.9%) | **p-value** |
| --- | --- | --- | --- | --- |
| Age (years) | 64.4 ± 15.6 | 69.2 ± 12.6 | 63.2 ± 16.0 | 0.03 |
| Male – N (%) | 142 (67.9) | 26 (59.1) | 116 (70.3) | 0.16 |
| BMI (kg / m^2^)^a^ | 27.5 ± 6.7 | 28.4 ± 7.7 | 27.2 ± 6.4 | 0.55 |
| Non-pulmonary SOFA (adjusted for missing values) ^b^ | 5.8 ± 3.9 | 5.5 ± 3.6 | 5.9 ± 4.0 | 0.59 |
| **ARDS Risk factors – N (%)** |  |  |  | 0.94 |
| No risk factors | 19 (9.1) | 4 (9.1) | 15 (9.1) |  |
| Non-pulmonary risk factors | 41 (19.6) | 9 (20.5) | 32 (19.4) |  |
| Pulmonary risk factors | 114 (54.5) | 25 (56.8) | 89 (53.9) |  |
| Pulmonary and non-pulmonary risk factors | 35 (16.7) | 6 (13.6) | 29 (17.6) |  |
| **Co-morbidities – N (%)** |  |  |  |  |
| COPD or home ventilation | 52 (24.9) | 13 (29.5) | 39 (23.6) | 0.42 |
| Chronic cardiac failure (NYHA III-IV) | 24 (11.4) | 7 (15.9) | 17 (10.3) | 0.30 |
| Chronic liver failure (Child-Pugh C) | 6 (2.3) | 2 (4.5) | 4 (2.4) | 0.45 |
| Chronic renal failure | 20 (9.6) | 8 (18.2) | 12 (7.3) | 0.03 |
| Active Neoplasm | 26 (12.4) | 4 (9.1) | 22 (13.3) | 0.45 |
| Immunosuppression | 12 (5.7) | 2 (4.5) | 10 (6.1) | 0.70 |
| Haematologic neoplasm | 4 (1.9) | 0 (0) | 4 (2.4) | 0.30 |
| Active Neoplasm / Immunosuppression / Haematologic Neoplasm | 37 (17.7) | 5 (11.4) | 32 (19.4) | 0.21 |
| **Ventilator variables** |  |  |  |  |
| PaO_2_:FiO_2_ ­ratio (mmHg)^c^ | 175.1 ± 71.6 | 180.0 ± 69.1 | 173.7 ± 72.4 | 0.59 |
| Tidal Volume (ml / kg PBW) ^d^ | 8.0 ± 2.1 | 8.3 ± 2.3 | 8.0 ± 2.0 | 0.60 |
| Plateau pressure (cmH_2_O)^e^ | 20.7 ± 4.9 | 22.6 ± 3.6 | 20.2 ± 5.1 | 0.13 |
| PEEP (cmH_2_O)^f^ | 7.2 ± 2.6 | 7.1 ± 2.4 | 7.2 ± 2.7 | 0.95 |
| Peak inspiratory pressure (cmH_2_O)^g^ | 25.0 ± 8.8 | 27.3 ± 9.8 | 24.4 ± 8.4 | 0.06 |
| Respiratory rate (breaths / min)^h^ | 20.1 ± 6.3 | 20.5 ± 6.9 | 19.9 ± 6.1 | 0.78 |
| Blood pH^i^ | 7.34 ± 0.11 | 7.34 ± 0.11 | 7.34 ± 0.11 | 0.74 |
| PaCO2 (mmHg)^j^ | 45.6 ± 16.2 | 45.8 ± 12.7 | 45.6 ± 17.0 | 0.39 |
| Bicarbonate (mmol / L)^j^ | 23.4 ± 5.8 | 23.5 ± 5.1 | 23.3 ± 6.0 | 0.83 |

Values reported as mean ± standard deviation unless otherwise stated.

Abbreviations: ARDS: acute respiratory distress syndrome; BMI: body mass index; COPD: chronic obstructive pulmonary disease, NYHA: New York heart association functional classification; PBW: predicted body weight; PEEP: positive end-expiratory pressure; SOFA: sequential organ failure assessment.

A: Measured in 44 diabetics, 156 non-diabetics; B: Measured in 44 diabetics, 164 non-diabetics; C: Measured in 44 diabetics, 160 non-diabetics; D: Measured in 40 diabetics, 138 non-diabetics; E: Measured 12 diabetics, 47 non-diabetics; F: Measured in 44 diabetics, 150 non-diabetics; G: Measured in 40 diabetics, 150 non-diabetics; H: Measured in 43 diabetics, 155 non-diabetics; I: Measured in 44 diabetics, 161 non-diabetics; J: Measured in 44 diabetics, 161 non-diabetics.

**Table S3: Univariate analysis for developing ARDS after day 2**

| **Variable** | **Odds ratio (95% CI)** | **p-value** |
| --- | --- | --- |
| Diabetes diagnosis | 0.86 (0.60, 1.24) | 0.43 |
| Age (years) | 1.008 (0.999, 1.018) | 0.08 |
| Male | 1.28 (0.94, 1.76) | 0.12 |
| BMI (kg / m^2^)^a^ | 1.00 (0.98, 1.02) | 0.68 |
| **Risk factor for ARDS** |  |  |
| Pulmonary | 1.90 (1.13, 3.18) | 0.01 |
| Non-pulmonary | 1.38 (0.78, 2.46) | 0.27 |
| Pulmonary and non-pulmonary | 2.39 (1.30, 4.36) | 0.005 |
| **Chronic disease** |  |  |
| Chronic cardiac failure (NYHA III-IV) | 1.11 (0.69, 1.76) | 0.68 |
| Chronic liver failure (Child-Pugh C) | 0.72 (0.30, 1.71) | 0.45 |
| Chronic renal failure | 0.84 (0.51, 1.37) | 0.48 |
| Active Neoplasm | 1.26 (0.80, 1.99) | 0.32 |
| Haematologic neoplasm | 0.74 (0.26, 2.12) | 0.57 |
| Immunosuppression | 0.69 (0.37, 1.29) | 0.24 |
| Active neoplasm / haematologic neoplasm / immunosuppression | 0.96 (0.65, 1.41) | 0.83 |
| COPD or home ventilation | 0.84 (0.60, 1.19) | 0.33 |
| **Ventilator variables at baseline** |  |  |
| PaO_2_:FiO_2_ ratio (mmHg)^b^ | 0.997 (0.995, 0.999) | 0.009 |
| tidal volume (ml/kg PBW) ^c^ | 1.03 (0.96, 1.09) | 0.45 |
| Plateau pressure at diagnosis (cmH_2_O)^d^ | 1.00 (0.95, 1.06) | 0.84 |
| Non-pulmonary SOFA (adjusted for missing values) ^e^ | 1.00 (0.96, 1.03) | 0.80 |
| PEEP (cmH_2_O)^f^ | 1.02 (0.97, 1.08) | 0.42 |
| Peak inspiratory pressure (cmH_2_O)^g^ | 1.03 (1.01, 1.05) | 0.004 |
| Respiratory rate (breaths / min)^h^ | 1.00 (0.98, 1.03) | 0.76 |
| Blood pH^i^ | 0.45 (0.13, 1.57) | 0.21 |
| PaCO_2_ (mmHg)^j^ | 1.00 (0.99, 1.01) | 0.45 |
| Bicarbonate (mmol / L)^j^ | 0.99 (0.97, 1.01) | 0.35 |

Abbreviations: ARDS: acute respiratory distress syndrome; BMI: body mass index; CI: confidence interval; COPD: chronic obstructive pulmonary disease, NYHA: New York heart association functional classification; PBW: predicted body weight; PEEP: positive end-expiratory pressure; SOFA: sequential organ failure assessment.

Analysis based on data from 1294 observations (unless otherwise stated)

A: Data from 1216 observations; B: Data from 1286 observations; C: Data from 1138 observations; D: Data from 291 observations; E: Data from 1282 observations; F: Data from 1268 observations; G: Data from 1194 observations; H: Data from 1270 observations; I: Data from 1270 observations; J: Data from 1270 0bservations.

**Table S4: Patient outcomes from ARDS that developed after day 2**

| **Outcome** | **Overall**  (n = 209) | **Diabetic**  (N = 44) | **Non-diabetic**  (N = 165) | **P-value** |
| --- | --- | --- | --- | --- |
| Duration of invasive mechanical ventilation (days)^a^ | 13 (8 – 20.5) | 14 (8 – 21) | 13 (8 – 20) | 0.95 |
| Duration of invasive mechanical ventilation in survivors (at hospital discharge) (days)^b^ | 13 (8 – 23) | 12 (8 – 31) | 13 (8 – 22) | 0.89 |
| Duration of invasive mechanical ventilation in non-survivors (at hospital discharge) (days)^c^ | 13 (8 – 20) | 15.5 (9.5 – 19) | 11 (7 – 20) | 0.59 |
| Hospital mortality ^*^ – N (%) | 78 (37.3) | 18 (40.9) | 60 (36.4) | 0.58 |

Values reported as median (interquartile range) unless otherwise stated.

* Includes patients who received non-invasive mechanical ventilation only.

A: N = 176, 37 diabetic, 139 non-diabetic; B: N = 103, 21 diabetic, 82 non-diabetic; C: N = 73, 16 diabetic, 57 non-diabetic.

**Table S5: Univariate analysis of factors associated with having ARDS**

| **Variable** | **Odds ratio (95% CI)** | **p-value** |
| --- | --- | --- |
| Diabetes diagnosis | 0.90 (0.76 – 1.06) | 0.21 |
| Age (years) | 0.997 (0.993 – 1.002) | 0.24 |
| Male | 0.98 (0.85 – 1.14) | 0.82 |
| BMI (kg / m^2^)^a^ | 1.004 (0.995 – 1.014) | 0.34 |
| Non-pulmonary SOFA score (adjusted for missing values)^b^ | 1.02 (1.00 – 1.03) | 0.10 |
| **ARDS Risk factors** |  |  |
| Pulmonary | 2.21 (1.78 – 2.75) | <0.0001 |
| Non-Pulmonary | 1.45 (1.13 – 1.85) | 0.003 |
| Pulmonary and Non-pulmonary | 2.20 (1.67 – 2.90) | <0.0001 |
| **Chronic disease** |  |  |
| Chronic cardiac failure (NYHA III-IV) | 0.99 (0.479 – 1.24) | 0.91 |
| Chronic liver failure | 0.99 (0.69 – 1.41) | 0.93 |
| Chronic renal failure | 0.89 (0.71 – 1.11) | 0.30 |
| COPD or home ventilation | 0.74 (0.64 – 0.87) | 0.0002 |
| Active neoplasm | 0.83 (0.65 – 1.05) | 0.11 |
| Immunosuppression | 1.56 (1.22 – 1.99) | 0.0004 |
| Haematologic neoplasm | 1.86 (1.23 – 2.81) | 0.003 |
| Active neoplasm / Immunosuppression / Haematologic neoplasm | 1.15 (0.96 – 1.38) | 0.12 |
| **Ventilator variables at baseline** |  |  |
| PaO_2_:FiO_2_ ratio (mmHg)^c^ | 0.994 (0.993 – 0.995) | <0.0001 |
| Tidal volume (ml/kg PBW)^d^ | 0.97 (0.94 – 1.00) | 0.08 |
| Plateau pressure (cmH_2_O)^e^ | 1.08 (1.05 – 1.11) | <0.0001 |
| PEEP (cmH_2_O)^f^ | 1.14 (1.11 – 1.17) | <0.0001 |
| Peak inspiratory pressure (cmH_2_O)^g^ | 1.03 (1.02 – 1.04) | <0.0001 |
| Respiratory rate (breaths / min)^h^ | 1.04 (1.03 – 1.05) | <0.0001 |
| Blood pH^i^ | 0.25 (0.14 – 0.47) | <0.0001 |
| PaCO_2_ (mmHg)^j^ | 1.01 (1.00 – 1.01) | 0.01 |
| Bicarbonate (mmol / L) | 0.99 (0.98 – 1.00) | 0.10 |

Abbreviations: ARDS: acute respiratory distress syndrome; BMI: body mass index; CI: confidence interval; COPD: chronic obstructive pulmonary disease, NYHA: New York heart association functional classification; PBW: predicted body weight; PEEP: positive end-expiratory pressure; SOFA: sequential organ failure assessment.

A: Data from 3882 observations; B: Data from 4076 observations; C: Data from 4086 obsevations; D: Data from 3670 observations; E: Data from 1004 obsersvations; F: Data from 4073 observations; G: Data from 3849 observations; H: Data from 4068 observations; I: Data from 4042 obsevations; J: Data from 4040 observations; K: Data from 4039 observations.

**Table S6: Baseline characteristics of patients with ARDS.**

| **Variable** | **Overall**  (n = 3022) | **Diabetics**  (n = 657; 21.7%) | **Non-diabetics**  (n = 2365; 78.3%) | **p-value** |
| --- | --- | --- | --- | --- |
| Age (years) | 61.5 ± 16.7 | 66.9 ± 13.3 | 60.0 ± 17.3 | <0.0001 |
| Male – N (%) | 1871 (61.9) | 414 (63.0) | 1457 (61.6) | 0.51 |
| BMI (kg / m^2^)^a^ | 27.5 ± 8.6 | 30.4 ± 12.5 | 26.7 ± 6.9 | <0.0001 |
| Baseline Non-pulmonary SOFA adjusted for missing values ^b^ | 6.2 ± 4.0 | 6.3 ± 4.1 | 6.1 ± 4.0 | 0.67 |
| **ARDS Risk factors – N (%)** |  |  |  | 0.08 |
| No risk factors | 253 (8.4) | 69 (10.5) | 184 (7.8) |  |
| Non-pulmonary risk factors | 572 (18.9) | 114 (17.4) | 458 (19.4) |  |
| Pulmonary risk factors | 1767 (58.5) | 389 (59.2) | 1378 (58.3) |  |
| Pulmonary and non-pulmonary risk factors | 430 (14.2) | 85 (12.9) | 345 (14.6) |  |
| **Co-morbidities – N (%)** |  |  |  |  |
| No co-morbidity other than diabetes | 1455 (48.1) | 250 (38.1) | 1205 (51.0) | <0.0001 |
| COPD or home ventilation | 683 (22.6) | 206 (31.4) | 477 (20.2) | <0.0001 |
| Chronic cardiac failure (NYHA III-IV) | 314 (10.4) | 120 (18.3) | 194 (8.2) | <.0001 |
| Chronic liver failure (Child-Pugh C) | 118 (3.9) | 29 (4.4) | 89 (3.8) | 0.45 |
| Chronic renal failure | 306 (10.1) | 151 (23.0) | 155 (6.6) | <0.0001 |
| Active Neoplasm | 258 (8.5) | 40 (6.1) | 218 (9.2) | 0.01 |
| Immunosuppression | 365 (12.1) | 58 (8.8) | 307 (13.0) | 0.004 |
| Haematologic neoplasm | 142 (4.7) | 16 (2.4) | 126 (5.3) | 0.001 |
| Active Neoplasm / Immunosuppression / Haematologic Neoplasm | 621 (20.5) | 95 (14.5) | 526 (22.2) | <0.0001 |
| **Ventilator variables at baseline** |  |  |  |  |
| PaO_2_:FiO_2_ ­ratio (mmHg)^c^ | 160.1 ± 67.9 | 163.0 ± 67.7 | 159.3 ± 67.9 | 0.19 |
| Tidal Volume (ml / kg PBW) ^d^ | 7.8 ± 2.1 | 7.9 ± 2.1 | 7.7 ± 2.1 | 0.04 |
| Plateau pressure (cmH_2_O)^e^ | 22.9 ± 6.0 | 24.3 ± 5.2 | 22.5 ± 6.2 | 0.0004 |
| PEEP (cmH_2_O)^f^ | 8.1 ± 3.2 | 8.0 ± 3.1 | 8.1 ± 3.2 | 0.84 |
| Peak inspiratory pressure (cmH_2_O)^g^ | 25.4 ± 8.9 | 25.9 ± 8.9 | 25.3 ± 8.9 | 0.12 |
| Respiratory rate (breaths / min)^h^ | 21.7 ± 8.6 | 21.3 ± 7.1 | 21.8 ± 8.9 | 0.15 |
| Blood pH ^i^ | 7.33 ± 0.12 | 7.32 ± 0.13 | 7.33 ± 0.12 | 0.05 |
| PaCO_2_ (mmHg)^j^ | 46.1 ± 15.9 | 46.4 ± 15.9 | 46.1 ± 15.9 | 0.53 |
| Bicarbonate (mmol / L)^k^ | 23.5 ± 6.8 | 23.3 ± 6.9 | 23.5 ± 6.7 | 0.28 |

Values reported as mean ± standard deviation unless otherwise stated.

Abbreviations: ARDS: acute respiratory distress syndrome; BMI: body mass index; COPD: chronic obstructive pulmonary disease, NYHA: New York heart association functional classification; PBW: predicted body weight; PEEP: positive end-expiratory pressure; P/F: partial pressure of arterial blood oxygen content to inspired fraction of oxygen; SOFA: sequential organ failure assessment.

A Measured in 632 diabetics, 2234 non-diabetics.; B Measured in 654 diabetics, 2348 non-diabetics; C Measured in 655 diabetics, 2349 non-diabetics; D Measured in 595 diabetics, 2115 non-diabetics; E Measured in 176 diabetics, 596 non-diabetics; F Measured in 655 diabetics, 2343 non-diabetics; G Measured in 622 diabetics, 2223 non-diabetics; H Measured in 654 diabetics, 2342 non-diabetics; I Measured in 647 diabetics, 2330 non-diabetics; K Measured in 645 diabetics, 2329 non-diabetics.

**Table S7: Patient outcomes from ARDS.**

| **Outcome** | **Overall**  (n = 3022) | **Diabetic**  (N = 657) | **Non-diabetic**  (N = 2365) | **P-value** |
| --- | --- | --- | --- | --- |
| Duration of invasive mechanical ventilation (days)^a^ | 8 (4 – 16) | 9 (4 – 16) | 8 (4 – 16) | 0.70 |
| Duration of invasive mechanical ventilation in survivors (at hospital discharge) (days)^b^ | 9 (4 – 16) | 8 (4 – 16) | 9 (4 – 15) | 0.98 |
| Duration of invasive mechanical ventilation in non-survivors (at hospital discharge) (days)^c^ | 8 (4 – 16) | 9 (4 – 16) | 8 (4 – 16) | 0.55 |
| Hospital mortality ^d*^ – N (%) | 1187 (39.4) | 272 (41.6) | 915 (38.8) | 0.19 |
| Hospital mortality in patients with only pulmonary risk factors ^e^ – N (%) | 694 (39.4) | 165 (42.6) | 529 (38.5) | 0.14 |
| Hospital mortality in patients with only non-pulmonary risk factors ^f^ – N (%) | 218 (38.2) | 35 (31.0) | 183 (40.0) | 0.08 |

Values reported as median (interquartile range) unless otherwise stated.

* Includes patients who received non-invasive mechanical ventilation only.

A: N = 2566, 548 diabetic, 2018 non-diabetic.; B: N = 1472, 299 diabetic, 1173 non-diabetic; C: N = 1086, 246 diabetic, 840 non-diabetic; D: data missing in 9 subjects (3 diabetic, 6 non-diabetic); E: N = 1760, 387 diabetic, 1373 non-diabetic; F: N = 571, 113 diabetic, 458 non-diabetic.

**Table S8: Univariate analysis for hospital mortality in patients with ARDS**

| **Variable** | **Odds ratio (95% CI)** | **p-value** |
| --- | --- | --- |
| Diabetes diagnosis | 1.12 (0.94, 1.34) | 0.19 |
| Age (years) | 1.021 (1.016, 1.025) | <0.0001 |
| Male | 1.02 (0.87, 1.18) | 0.85 |
| BMI (kg / m^2^)^a^ | 0.97 (0.96, 0.98) | <0.0001 |
| Non-pulmonary SOFA score (adjusted for missing values)^b^ | 1.12 (1.10, 1.15) | <0.0001 |
| **ARDS Risk factors** |  |  |
| Pulmonary | 1.22 (0.93, 1.61) | 0.16 |
| Non-Pulmonary | 1.16 (0.85, 1.58) | 0.35 |
| Pulmonary and Non-pulmonary | 1.45 (1.05, 2.00) | 0.02 |
| **Co-morbidities** |  |  |
| Chronic renal failure | 1.45 (1.14, 1.84) | 0.002 |
| Chronic cardiac failure (NYHA III-IV) | 1.44 (1.14, 1.83) | 0.002 |
| Chronic liver failure | 3.85 (2.57, 5.75) | <0.0001 |
| COPD or home ventilation | 0.96 (0.81, 1.14) | 0.65 |
| Active neoplasm | 1.82 (1.41, 2.36) | <0.0001 |
| Immunosuppression | 1.84 (1.48, 2.30) | <0.0001 |
| Haematologic neoplasm | 4.31 (2.96, 6.28) | <0.0001 |
| Active neoplasm / Immunosuppression / Haematologic neoplasm | 1.96 (1.64, 2.35) | <0.0001 |
| **Ventilator variables at baseline** |  |  |
| PaO_2_:FiO_2_ ratio (mmHg)^c^ | 0.998 (0.997, 0.999) | 0.0002 |
| Tidal volume (ml / kg PBW)^d^ | 0.99 (0.95, 1.02) | 0.45 |
| Plateau pressure (cmH_2_O)^e^ | 1.04 (1.01, 1.06) | 0.002 |
| PEEP (cmH_2_O)^f^ | 0.99 (0.97, 1.02) | 0.46 |
| Peak inspiratory pressure (cmH_2_O) ^g^ | 1.01 (1.01, 1.02) | 0.002 |
| Respiratory rate (breaths / min)^h^ | 1.02 (1.01, 1.03) | 0.0002 |
| Blood pH^i^ | 0.09 (0.05, 0.17) | <0.0001 |
| PaCO_2_ (mmHg)^j^ | 1.00 (0.99, 1.00) | 0.29 |
| Bicarbonate (mmol / L)^k^ | 0.95 (0.94, 0.97) | <0.0001 |

Abbreviations: ARDS: acute respiratory distress syndrome; BMI: body mass index; CI: confidence interval; COPD: chronic obstructive pulmonary disease, NYHA: New York heart association functional classification; PBW: predicted body weight; PEEP: positive end-expiratory pressure; SOFA: sequential organ failure assessment.

Analysis based on data from 3013 observations (unless otherwise stated)

A: Data from 2857 observations; B: Data from 2993 observations; C: Data from 2995 observations; D: Data from 2701 observations; E: Data from 768 observations; F: Data from 2989 observations; G: Data from 2837 observations; H: Data from 2987 observations; I: Data from 2968 observations; J: Data from 2966 observations; K: Data from 2965 observations.

**Table S9: Univariate analysis for hospital mortality in patients who developed ARDS after day 2**

| **Variable** | **Odds ratio (95% CI)** | **p-value** |
| --- | --- | --- |
| Diabetes diagnosis | 1.21 (0.61, 2.39) | 0.58 |
| Age (years) | 1.03 (1.01, 1.05) | 0.008 |
| Male | 0.83 (0.46, 1.51) | 0.54 |
| BMI (kg / m^2^) | 0.98 (0.93, 1.02) | 0.28 |
| **Ventilator variables at baseline** |  |  |
| PaO_2_:FiO_2_ ratio (mmHg)^a^ | 1.00 (0.997, 1.005) | 0.60 |
| Tidal volume (ml/kg PBW) ^b^ | 1.02 (0.88, 1.18) | 0.80 |
| Plateau pressure (cmH_2_O)^c^ | 0.99 (0.89, 1.10) | 0.80 |
| Non-pulmonary SOFA adjusted for missing values ^d^ | 1.06 (0.99, 1.14) | 0.10 |
| Total respiratory rate (breaths / min)^e^ | 0.94 (0.89, 0.99) | 0.01 |
| Blood pH^f^ | 3.05 (0.23, 40.52) | 0.40 |
| PaCO_2_ (mmHg)^f^ | 0.99 (0.97, 1.01) | 0.25 |
| Bicarbonate (mmol / L)^f^ | 0.98 (0.93, 1.03) | 0.41 |
| **Risk factor for ARDS** |  |  |
| Pulmonary | 1.33 (0.47, 3.81) | 0.59 |
| Non-pulmonary | 1.23 (0.39, 3.93) | 0.73 |
| Pulmonary and non-pulmonary | 0.83 (0.24, 2.84) | 0.77 |
| **Co-morbidities** |  |  |
| Chronic cardiac failure (NYHA III-IV) | 2.18 (0.93, 5.14) | 0.07 |
| Chronic liver failure (Child-Pugh C) | 0.84 (0.15, 4.67) | 0.84 |
| Chronic renal failure | 0.90 (0.34, 2.35) | 0.82 |
| Active Neoplasm | 1.82 (0.80, 4.15) | 0.16 |
| Haematologic neoplasm | 5.2 (0.53, 50.89) | 0.16 |
| Immunosuppression | 3.63 (1.06, 12.5) | 0.04 |
| Active neoplasm / haematologic neoplasm/ immunosuppression | 2.31 (1.13, 4.75) | 0.02 |
| COPD or home ventilation | 1.07 (0.56, 2.04) | 0.84 |

Abbreviations: ARDS: acute respiratory distress syndrome; BMI: body mass index; CI: confidence interval; COPD: chronic obstructive pulmonary disease, NYHA: New York heart association functional classification; PBW: predicted body weight; PEEP: positive end-expiratory pressure; SOFA: sequential organ failure assessment.

Analysis based on data from 209 observations (unless otherwise stated).

A: 5 missing observations; B: 31 missing observations; C: 150 missing observations; D: 1 missing observation.; E: 11 missing observations; F: 4 missing observations.

**Table S10: Multivariable analysis for hospital mortality in patients who developed ARDS after day 2**

| **Variable** | **Odds ratio (95% CI)** | **p-value** |
| --- | --- | --- |
| Diabetes diagnosis | 1.07 (0.53, 2.17) | 0.84 |
| Age (years) | 1.03 (1.01, 1.05) | 0.008 |
| Immunosuppression | 3.89 (1.11, 13.61) | 0.03 |

Analysis based on data from 209 observations

Abbreviations: CI: confidence interval.
